# Comparison of genetic variants in matched samples using thesaurus annotation

**DOI:** 10.1093/bioinformatics/btv654

**Published:** 2015-11-05

**Authors:** Tomasz Konopka, Sebastian M.B. Nijman

**Affiliations:** Ludwig Institute for Cancer Research, University of Oxford, Oxford, UK

## Abstract

**Motivation:** Calling changes in DNA, e.g. as a result of somatic events in cancer, requires analysis of multiple matched sequenced samples. Events in low-mappability regions of the human genome are difficult to encode in variant call files and have been under-reported as a result. However, they can be described accurately through thesaurus annotation—a technique that links multiple genomic loci together to explicate a single variant.

**Results:** We here describe software and benchmarks for using thesaurus annotation to detect point changes in DNA from matched samples. In benchmarks on matched normal/tumor samples we show that the technique can recover between five and ten percent more true events than conventional approaches, while strictly limiting false discovery and being fully consistent with popular variant analysis workflows. We also demonstrate the utility of the approach for analysis of *de novo* mutations in parents/child families.

**Availability and implementation:** Software performing thesaurus annotation is implemented in java; available in source code on github at GeneticThesaurus (https://github.com/tkonopka/GeneticThesaurus) and as an executable on sourceforge at geneticthesaurus (https://sourceforge.net/projects/geneticthesaurus). Mutation calling is implemented in an R package available on github at RGeneticThesaurus (https://github.com/tkonopka/RGeneticThesaurus).

**Supplementary information:** Supplementary data are available at *Bioinformatics* online.

**Contact:** tomasz.konopka@ludwig.ox.ac.uk

## 1 Introduction

Variants and mutations in DNA underlie several human diseases. Studies of whole-exome and whole-genome datasets based on short-read shotgun sequencing have already uncovered much information about the mutational landscape in disease. This is exemplified by the growth of impressive catalogues of genetic markers linked to Mendelian inherited conditions (Hamosh *etal*., 2005) and to cancer (Forbes *etal*., 2014). However, considerable portions of the human genome consist of non-unique sequences (Treangen *etal*., 2011), which have posed challenges for bioinformatic analyses and for interpretations of findings. Thus, the mutational landscape in these regions still remains concealed.

To identify non-germline genetic features linked with a disease, most mutation calling workflows process several matched samples at the same time (Pabinger *etal*., 2013). For example, somatic mutation callers for cancer genomics consider a tumor sample together with a normal control from the same individual. One sample (the tumor) is used to put forth a list of candidate sites that are different than in the reference genome. Information from matched control samples (normal tissue) is then used to eliminate features that also appear in cells unaffected by the disease. The control sample is thus used for personalized filtering of the candidate list. Because common features in case and control samples may arise due to the germ-line or because of sequencing artifacts, it is tempting to use models or databases of polymorphisms for this step. However, despite increasing detail in the databases and despite increasing understanding of sequencing chemistry, the personalized filtering strategy remains the most effective means to identify disease-associated variants (Jones *etal*., 2015).

Some approaches for personalized filtering are threshold- and rule-driven (Koboldt *etal*., 2012; Larson *etal.*, 2012; Roth *etal.* 2012), while others emphasize Bayesian models (Cibulskis *etal*., 2013; Rimmer *etal*., 2014; Saunders *et al.*, 2012). The best approach is difficult to pin down because cancer genomes are heterogeneous, contain regions of unusual copy number, and because tumor samples are often mixtures of cancer and non-cancer cells; approaches have been designed to handle these possibilities to various extent. Indeed, tuning settings for individual workflows is still an active area of discussion (Ewing *etal*., 2015). Moreover, as sequencing technology is applied in niche settings, specialized software is often required, for example for detecting ultra-low frequency mutations (e.g. He *etal*., 2015) or analyzing sample groups with several matched samples (Josephidou *etal*., 2015). A separate branch of the literature avoids idiosyncrasies of cancer samples altogether and focuses on family trios (e.g. Li *et al.*, 2012; Ramu *et al.*, 2013; Wei *etal*., 2015).

Mutations are usually reported using coordinates in a reference genome, i.e. in variant call files (VCF). For example, the common BRAF mutation V600E in cancer patients is described as ‘genome hg38, position chr7:140,753,336, substitution A > T.’ This approach is effective in most cases, but it is fundamentally unsuited for describing variation in low-mappability regions. When reads measured via high-throughput sequencing align onto multiple areas of a reference genome, it is impossible to assign a mutation unambiguously to a single locus. As a result, genetic variation in such regions can go unreported. False negatives may constitute 10% of the total genetic variation in a human sample. Thus mappability is sometimes described as the leading outstanding problem for variant calling (Ewing *etal*., 2015).

A complementary approach to reporting single-nucleotide variants, called the thesaurus approach, is to link candidate sites in low-mappability regions to alternative loci (Kerzendorfer *etal*., 2015). In this approach, links between pairs of sites suggest that they are synonymous as far as alignment is concerned. By symmetry and transitivity, genomic sites self-organize into clusters (if site A is linked to site B, then sites A and B form a cluster; if site A is linked to site B and site B is linked to site C, then sites A, B and C form a cluster; and so on). These clusters, grounded on more than one genomic coordinate, then constitute the basis for comparisons, interpretations and any follow-up analysis. A software implementation of this approach (GeneticThesaurus v0.1) has been shown to improve sensitivity in calling germline variation in single samples, maintaining a manageable false discovery rate. Importantly, variants in low mappability regions have been shown to lie in genomic regions that are functionally relevant to diseases (i.e. exons of coding genes, regulatory elements, etc.). They have also been shown to be experimentally reproducible with alternative sequencing methods (capillary sequencing).

In this work, we extend the thesaurus approach to enhance detection of DNA changes across matched samples. In other words, we implement a personalized filtering strategy taking thesaurus annotations into account. This contribution removes low mapping quality from the list of difficulties in the analysis of matched sample and thus enables, for the first time, to use short-read sequencing data to describe the landscape of mutations in sequence-similar regions of the human genome. The implementation is designed to be general-purpose and extensible in order to accommodate several use-cases, in particular the genomics of cancer and of familial diseases.

## 2 Methods

Our contribution in this work has two distinct parts. The first is a new release of the GeneticThesaurus software (v0.2) adapted to the multiple sample setting. The second is a separate package to help the exploration of thesaurus annotations in the R environment (R Core Team, 2014). Before describing these contributions, however, we first briefly review the thesaurus annotation scheme.

### 2.1 Thesaurus annotation in single samples

**Fig. 1.**
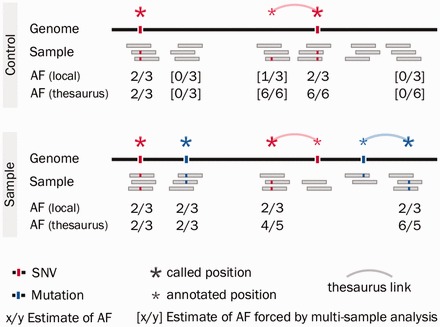
Overview of thesaurus annotation of variants in a sample/control pair. On top, a control genome contains two germline variants: one in a high- (left) and one in a low-mappability region (right). Reads with evidence for the latter variant are distributed over two sites. On bottom, a related genome contains the same germline variants and two mutations. Thesaurus clusters of variants accurately capture the true features in the samples. Allelic frequency (AF) estimates are shown for each variant

The thesaurus annotation approach is illustrated in Figure 1. When a genetic variant is located in a unique region of the genome, sequenced reads align correctly and evidence for a mismatch accumulates at the variant site (Fig. 1, left). In such cases, the allelic frequency of the variant can be estimated by the ratio of reads exhibiting a mismatch relative to the total coverage at the locus. We call this a ‘local’ allelic frequency and compute it on a site *i* as

(1) $${\left[AF\right]}_{local}(i)=m(i)/C(i),$$

where *m*(*i*) is the number of reads containing the mismatch and *C*(*i*) is the total coverage.

When a variant in located in a low mappability region of the genome, short sequenced reads cannot be aligned unambiguously. Evidence for mismatches are then distributed and diluted over multiple genomic sites; a local variant calling approach may detect any combination of these as candidate variants (Fig. 1, right). For such variants, thesaurus annotation provides links to related sites in the genome. Sometimes links may point from a false location to the correct site of the variant. At other times links may point to sites that are not the true site of the variant, but may contains misaligned reads containing evidence for the variant (Fig. 1, bottom). We use the thesaurus-linked sites to compute a thesaurus-adjusted allelic frequency for the called variants. If we denote by *T*(*i*) the set of all genomic sites linked to site *i* (including *i* itself), then the thesaurus allelic frequency is defined as

(2) $${\left[AF\right]}_{thesaurus}(i)=|T(i)|(\sum_{j\in T(i)}m(j))/(\sum_{k\in T(i)}C(k)),$$

where, as before, *m*(*i*) is the number of reads with a mismatch at site *i* and *C*(*i*) is the corresponding coverage. Examples of this counting method appear in the figure.

In terms of implementation, GeneticThesaurus analyzes an input alignment in two passes. In the first pass it scans an alignment and builds a set of thesaurus links for every variant candidate determined by an input VCF file. This process contains several steps and conditions to adjust thesaurus links based on the aligned reads (Kerzendorfer *etal.* 2015). In the second pass the software collects read count information from all the relevant genomic sites and computes the local and thesaurus-adjusted frequencies.

From the user perspective, the output of thesaurus annotation consists of three components (Other output data types, e.g. tables with cluster ids and database annotations of thesaurus links, can be obtained by running post-processing utilities on the primary output). The first component consists of a VCF table. Each VCF entry is amended with a tag to the format and genotype columns indicating the number of thesaurus links. When this number is nonzero, a VCF entry is also assigned an additional filter code thesaurus. Some sites are labeled with other filter codes: thesaurusmany when a site can be linked to an excessive number of alternative sites, or thesaurushard when a site is inside a thesaurus-annotated region but whose partners cannot be computed, e.g. because of surrounding indels.

The second component of the thesaurus output is a dedicated file enumerating all the relevant alternative sites. This information could in principle be added into the VCF file itself. However, this approach would require major changes to the VCF table, including adding new lines and filling in all columns of the VCF table, for which formats may vary across different workflows. In order to minimize the possibilities of conflicts, the information is instead kept separate.

The third component of the output is a table of allelic frequencies evaluated for each called variant using naive read counting as well as using the thesaurus-adjusted formula.

### 2.2 Allele frequencies from matched samples

The extension of the GeneticThesaurus software to matched samples lies in the second pass of the annotation procedure.

The program starts the analysis in the same way as above: it builds a network of links for all variant candidates identified in the tumor sample and then scans the tumor sample again to collect allelic frequency estimates. But, next, it also scans all the matched samples using the same thesaurus link network. In this way, the output files from this analysis follow the same structure as in the single-sample analysis, but contain allelic information on all the candidate sites from all the matched samples. Compared to running a thesaurus annotation analysis separately on each sample, this gives additional information at key candidate sites. These additional allelic frequency estimates are marked in Figure 1.

It is worth stressing that the output of the thesaurus annotation program is not a final set of mutation calls. Rather, it is a representation of summary statistics on all candidate sites. This is akin to pileup information given by SAMtools (Li *etal*., 2009) and as such forms a foundation on which mutation calling frameworks can build on.

### 2.3 Mutation calls

We implemented a set of tools for manipulating thesaurus annotation in an R package RGeneticThesaurus. Among the available functions in the package are ones to import tables produced by the GeneticThesaurus software. These can be used to interface with other packages on variant annotation. They are also set to eliminate certain classes of variants by default: structural variants, and variants marked with filter codes thesaurusmany or thesaurushard. These functions can also be set to eliminate variants with other custom filter codes, for example strand bias.

The package provides rudimentary, threshold-based functions for calling mutations from allelic frequency data. One calls mutations under the following criteria: (i) the allelic frequency in the tumor sample is above a threshold (default 0.15); (ii) the fold change in allelic frequency between two samples is above a threshold (default 1.2); (iii) the allelic frequency in the matched control is below a threshold (default 0.05). Another function has similar requirements but is tailored to comparison of two matched samples in a family trio (child, mother, father) context.

Despite the naivety, the heuristic approach is useful because it is very transparent. The default settings select mutation candidates with strong allelic frequencies that can be distinguished from noise via Sanger sequencing. The functions are not intended to be the definitive solutions for analysis of all sample types. Indeed, specific experiments will likely require more complex treatment, possibly using additional information/assumptions (e.g. genomic copy number, sample purity, or pedigrees of the matched samples). Bayesian models are well suited for this purpose, but must be carefully formulated for each application.

### 2.4 Comparisons of call sets

To benchmark mutation calling performance, we introduce some terminology and quality metrics that are suitable to the context of thesaurus annotation. These metrics apply to calculations where a ground truth set of mutations is known, as is the case when working with synthetic data.

We denote sites in a ground truth dataset as actual positives [*AP*]. We denote mutation calls obtained from a workflow/method as candidates. We call a candidate that coincides with a position in the actual positives a true positive [*TP*]. A candidate site that is not an actual positive, but that is linked to an actual positive via a thesaurus link, is termed a thesaurus true positive [*TTP*]. A site within the actual positives that is not called (not in the set of true positives) and that is not linked to a called site (not in the set of thesaurus true positives) is termed as a false negative [*FN*]. A site within the call set that is neither an actual positive nor linked to an actual positive is a false positive [*FP*]. Because of the presence of thesaurus links, these definitions of [*FP*] and [*FN*] can be viewed as more ‘lenient’ than the usual definitions from the classification literature.

Using this terminology, we define the true positive rate (Note that this definition is slightly different from the ‘thesaurus true positive rate’ used in Kerzendorfer *et al.* (2015)) as

(3) $$[TPR]=\frac{\left[AP]-[FN\right]}{\left[AP\right]}.$$

Unlike the traditional formula [TPR]=[TP]/[AP], this definition avoids making reference to true positives. In a thesaurus annotated setting, it counts actual positives found through alternative sites as informative. Such hits increase the true positive rate by reducing the number of false negatives.

We define the false discovery rate as

(4) $$[FDR]=\frac{0.5+[FP]}{0.5+[FP]+[AP]-[FN]}.$$

This is a quantity normalized by the number of actual positives and calls made, so it is comparable across datasets. In contrast to the usual formula [FDR]=[FP]/([FP]+[TP]), we again avoid making reference to true positives. We also include a term 0.5 in both the numerator and the denominator to prevent [*FDR*] from ever falling to zero. This property will allow us to exhibit [*FDR*] on a logarithmic scale, even for experiments with zero false positives. However, for experiments with a very small number of true positives ([AP]−[FN]∼0), the constant can produce an artificially large [*FDR*] even with [FP]=0.

We emphasize again that these definitions for [*TPR*] and [*FDR*] are different from the usual ones in the literature. In principle they should be labeled as ‘thesaurus-adjusted-[*TPR*]’ and ‘thesaurus-adjusted-[*FDR*]’. However, we will use the shorter names for brevity. We will also henceforth omit the [·] notation.

## 3 Results and discussion

We applied the matched-sample thesaurus software on two synthetic datasets and performed a descriptive analysis of a whole genome family trio dataset.

### 3.1 Synthetic normal/tumor pairs

To measure the power to detect mutations in sample pairs, we generated synthetic data. We created a ‘normal’ genome by inserting single-nucleotide substitutions in the hg38 reference genome at a rate of one event per kilobase (rate $10^{-3}$ bp^– 1^). We then further mutated this genome at a rate 5 × 10^– 4^^ ^bp^– 1^ to emulate a matched ‘tumor.’ Such a high mutation rate is not characteristic of real cancers, but it creates many examples of somatic changes in a variety of genomics contexts, which is ideal for benchmarking. This setup is equivalent to a thousand repeats of simulations with a more realistic somatic mutation rate of 5 × 10^–^^7^^ ^bp^–^^1^.

Next, we created synthetic short-read data. We created one sample from the mock normal genome, one sample from the mock tumor genome, and samples mimicking normal-tumor mixtures. For each case, we extracted 2 × 100 bp reads with 400 bp inserts at regular intervals from the synthetic genomes (20 × coverage). The resulting datasets do not have realistic variability in coverage due to sampling, GC content biases, or other systematic effects. However, the generation procedure is transparent and reproducible. It also guarantees that all variants are well-represented in the data. This is useful for benchmarking, because discrepancies between expected and detected variants must be due to mappability alone.

We aligned samples onto the hg38 reference genome with Bowtie2 (Langmead *etal*., 2012) and called variants and mutations with Bamformatics (sourceforge.net/projects/bamformatics). We worked with stringent and lenient settings related to mapping quality, setting thresholds at 16 (MQ16) and 1 (MQ1), respectively. We annotated variants with thesaurus links and called somatic mutations at sites where the estimated allele frequencies differed by 0.2 between normal and tumor samples, with the frequency in normal sample below 0.05. We then compared calls—with and without thesaurus annotation—to the known mutation sites.

**Fig. 2.**
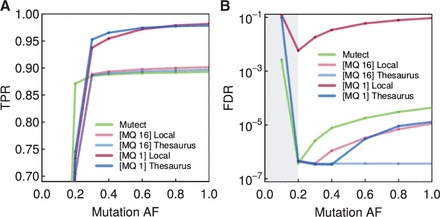
Performance for calling somatic mutations from synthetic normal/tumor sample pairs. (A) True positive rate (TPR) of various mutation detection approaches (note vertical axis does not start at 0 to emphasize the practically relevant TPR range). Dots represent synthetic tumor samples with different mutation allelic frequencies (AF). The approaches are Mutect, a local mutation calling at two mapping quality (MQ) thresholds, and thesaurus-assisted mutation calling at two mappability thresholds. Other methods are discussed in the Supplementary Text. (B) False discovery rates (FDR) for the same methods. The gray band indicates results with zero false positives and a small number of true positives, a performance regime that produces spuriously high FDRs based on Eq. (4)

When using a standard mappability threshold (MQ16) without thesaurus, recall of true mutations increased with mutation allele frequency and reached 90% in pure-tumor samples (Fig. 2A). The number of false positive calls was consistently low, indicating appropriate elimination of germline variants.

As expected, relaxing the mappability threshold (MQ1) increased recall, here to around 97% at high tumor content. The drawback of higher recall was the appearance of false positive calls (Fig. 2B). In samples with higher tumor allelic frequency, thousands of false positives raised FDR to close to 0.1, or one false call in ten. When we considered thesaurus annotation false discovery dropped substantially; notably at the MQ1 setting FDR fell to around 10^–^^5^, or one false call per hundred thousand calls. The same improvement was not reproduced by replacing the computed links with randomly generated links (Supplementary Text).

Benefits of thesaurus annotation were also observed relative to other mutation calling programs. Mutation calls by Mutect (Cibulskis *etal*., 2013) on the same datasets followed a similar pattern to what we described for stringent mappability settings without thesaurus (Fig. 2A and B). Mutation calls by Varscan2 (Koboldt *etal*., 2012) also followed the above results without thesaurus (Supplementary Text). Such similarities arise because the enhanced performance is due to linking related sites together, which is an innovation unique to the thesaurus approach.

We also performed thesaurus annotation calculations starting with variants from Platypus (Rimmer *etal*., 2014), an alternative caller, and observed similar improvement in performance (Supplementary Text). These observations suggest that the thesaurus implementation is not bound to a particular variant calling pipeline and can be incorporated into existing workflows.

### 3.2 Synthetic family trio

Next, we performed similar benchmarks for *de novo* mutation detection in a family trio. We created diploid genomes for a synthetic mother and father with random heterozygous variants inserted at a rate of 10^–^^3^^ ^bp^–^^1^. We then selected a set of chromosomes from each of the parents, mutated them further at a rate of 10^–^^4^^ ^bp^–^^1^, and treated the result as the genome of an offspring.

The calculations followed the same procedures as in the previous section. We generated synthetic reads for exactly 20× coverage of each genome, aligned them to the hg38 reference genome, called variants with two mapping quality settings, and then applied thesaurus multi-sample filtering. To search for *de novo* mutations in the child, we compared allelic frequencies in the child sample to the corresponding frequencies in both the parents’ samples. We evaluated candidates using a series of thresholds on the observed allelic frequency (AF).

**Fig. 3.**
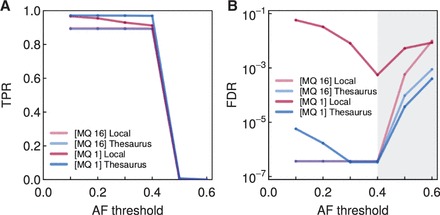
Performance for calling *de novo* mutations in a synthetic family trio. (A) True positive rates (TPR) of various calling approaches. Dots represent actual calls at various thresholds for observed allelic frequency (AF); lines are simple interpolations. The approaches used to select candidates are local mutation calling at two mapping quality (MQ) thresholds, and the thesaurus-assisted equivalents. (B) False discovery rates (FDR) for the same methods. The gray band indicates experiments with a small number of true positives and no false positives at all, for which Eq. (4) gives spuriously high FDR values

The majority of *de novo* variants in well-mappable regions were readily detectable in the child sample, with overall recall at around 90% (Fig. 3A). The true positive rate only fell when we required an allelic frequency above 0.5 (all the mutations were exactly heterozygous, hence none satisfied the excessive criterion). Including sites from low-mappability regions increased recall, but, as expected, substantially increased false discovery (Fig. 3B). However, thesaurus links resolved ambiguous mapping and reduced false discovery by orders of magnitude. At intermediate AF thresholds, thesaurus annotation also served to reduce the number of false negatives.

For our usual annotation pipeline, we allowed up to 100 thesaurus links per annotated site. In practice, however, many sites connected only to a handful of alternative loci. To illustrate this and at once test the robustness of the mutation calling procedure to the threshold on maximal links, we reran the annotation allowing at most ten links per site (Supplementary Text). As expected, this change increased the number of false negatives in the mutation call set. However, the overall shape of the TPR and FDR curves remained unaffected. Thus, the majority of the variants throughout the genome affected by mappability are in regions of relatively low copy number.

Finally, we observed that although the synthetic dataset contained *de novo* mutations with AF of exactly 0.5, some candidate sites received considerably smaller estimates (both naive and thesaurus adjusted). This implies that while reads with mismatches accumulated at these particular genomic loci (possibly because of idiosyncrasies of the aligner), the variants could not be linked to all their appropriate partners (possibly because of combinations of nearby variants or sub-optimal linking strategy). Missing links spoiled the adjustment for allelic frequency and failed to turn false positive sites into thesaurus true positives. Thus, additional thesaurus links would be required to decode the remaining variation in this trio.

### 3.3 Platinum family trio

**Fig. 4.**
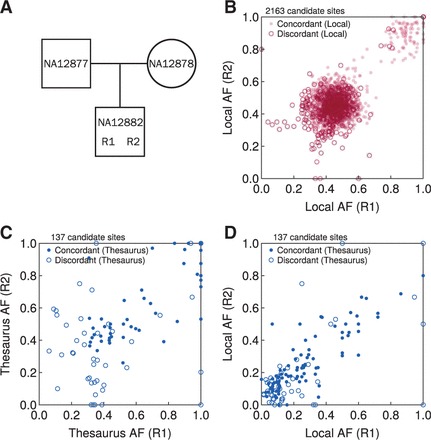
Results on *de novo* mutation calling from a family trio from the Platinum dataset. (A) Family pedigree for sample NA12882. R1 and R2 denote two replicates of the child’s genome. (B) Concordance of *de novo* mutation calls in two replicates of sample NA12882 obtained using a high (MQ 16) mapping quality threshold. Axes display the allelic frequencies (AF) of candidate sites in the two replicates. (C) Concordance of *de novo* mutation calls identified through thesaurus analysis of low mappability genomic regions (MQ 0) and not in the previous analysis. Allelic frequencies on the axes are informed by all thesaurus-linked genomic sites. (Thesaurus-adjusted AF > 1 are plotted at unity.) (D) Analogous to panel (C), but with allelic frequencies on the axes computed using data from only one genomic locus per mutation candidate

Following benchmarks on synthetic data, we turned to actual datasets that include a realistic mix of sequencing errors, coverage biases, insertions and deletion and other features. We obtained whole genome data for a family trio from the publicly available Platinum genomes project (Illimina, 2015). Although this dataset consists of a large family of three generations, we selected to work only with child NA12882 because DNA from this sample was sequenced in two independent replicates, which we denoted as R1 and R2 (Fig. 4A). In the absence of a ground truth set of mutations, we decided to explore consistency between these replicates instead.

The data from the Platinum genomes project had already been aligned with BWA (Li *etal*., 2009), so we used these ready alignments. We called variants in the NA12882 replicates using two mapping quality thresholds (MQ 0 and MQ 16). We then performed thesaurus multi-sample annotation using alignments from the child together with the parents (NA12877 and NA12878). To call *de novo* mutations, we set an allelic frequency threshold of 0.3 for variants in the child and a maximum allelic frequency of 0.02 in each of the parents. Because of increased error rate relative to the idealized synthetic datasets, we eliminated candidate sites supported by reads with excessive strand bias (Fisher test *P* < 0.06) and several mismatches (mean value of MN tag > 6). In calculations involving thesaurus annotation, we eliminated candidates marked with filter codes thesaurusmany and thesaurushard. We required a minimum coverage of 10 reads in each of the parents, except for candidates on chromosome Y, for which we applied this condition only in the father.

After processing each replicate separately, we compared the results. When working with variants identified at the higher mapping quality threshold, we obtained high concordance (Fig. 4B, Jaccard index 0.85). The number of mutation candidates—around 2000—was ten times higher than in reports of *de novo* mutations in primary patient samples (Kong *etal*., 2012), but was consistent with results on other trios in the Platinum dataset (Cleary *etal*., 2014), which are based on patient-derived cell lines. Mutations called in only one replicate, i.e. discordant sites, were often also present in the other, but not selected for the final mutation set because of lower allelic frequency or strand-bias filtering (strand-bias filtering falsely removes around 6% of true calls). These observations suggest that the sequencing data and the mutation calls are of high quality; the nominal value for concordance between replicates would likely increase with higher coverage and with additional technical replicates.

We then turned to mutations detected at the low mapping quality (Fig. 4C) and identified over 130 new mutation candidates using the thesaurus approach. The nominal concordance for called sites was lower than before (78 concordant sites, Jaccard index 0.57). However, the majority of candidate sites detected in one replicate also presented nonzero allelic frequencies in the other replicate (and not in the parents), albeit below threshold for selection to the final call set. This suggests that most of the identified sites are actually true, but appear discordant because of low coverage or an imperfect calling procedure. Considering borderline cases as actually concordant, the proportion of true variants in each replicate set could reach 80%, the previously reported experimental validation rate for thesaurus annotated variants (Kerzendorfer *etal*., 2015).

Because signal from a true variant is typically diluted over multiple sites, the effect of coverage on power to detect mutations is expected be more pronounced in low- than in high-mappability regions. To visualize this, we compared allelic frequencies computed using thesaurus-adjusted counting (Fig. 4C, Eq. (2)) and using local counting (Fig. 4D, Eq. (1)). Around two-thirds of concordant mutation candidate sites were identified because their thesaurus adjusted allelic frequencies satisfied our thresholds although their unadjusted counterparts did not. In other words, this group of reproducible variants was only called by collecting information from more than one site of the genome; identifying this group would not have been possible with any method considering only local information. Despite success in detecting these sites, the pronounced differences between local and thesaurus-adjusted allelic frequencies also highlight a limitation of the current implementation: because the initial mutation candidate set is guided by variants obtained with a standard variant caller, discrepancies due to low read counts can hinder analysis even prior to thesaurus link-building. Unfortunately the thesaurus software currently cannot rescue candidates missed in the initial screening stage.

While comparing mutation call sets, we also observed that around 50 candidates in the analysis based on high MQ candidates (Fig. 4B) were absent in the subsequent result sets. This can happen when a local AF indicates presence of a mutation but the thesaurus-adjusted AF fails the criteria. Some such sites may be germline contaminants that are eliminated by thesaurus annotation. Others can be attributed to filtering by thesaurusmany and thesaurushard codes, which eliminate sites cannot be reliably annotated with the current implementation of the thesaurus software.

Beyond comparisons of replicate datasets, the appropriate test for novel mutation candidates is independent validation in a separate dataset. In this direction, we analyzed a long-insert sequencing dataset based on the same family trio. In long-insert datasets, individual reads are still only 100 bp long, but are separated by longer gaps. The long-insert dataset for this family was lower in coverage than the primary dataset, so a direct comparison is not directly useful. However, we queried the long-insert dataset for the mutation sites of interest. We observed that the majority of the novel mutation candidates were also present in the long-insert data (Supplementary Text), reinforcing the view that a majority of the newly called variants are probably true. Of note, many of the new mutations remained supported only by reads with low mapping quality. This indicates that similarity of some mutation-containing genomic regions spans several kilobases where mapping ambiguities cannot be easily solved with long-insert sequencing.

Altogether, the thesaurus-based analysis puts forth more than 100 novel mutation sites in the NA12882 genome, an approximately 5% increase over the analysis using only high-mappability regions. This increase is comparable to, albeit slightly lower than, results using the synthetic datasets. Based on concordance between replicates, previous results on validation of thesaurus-annotated variants (Kerzendorfer *etal*., 2015), and in-silico validation in a long-insert dataset, we argued that the majority of the newly identified sites are likely to be true in the underlying biological sample.

## 4 Conclusion

In this work we extended the thesaurus variant annotation strategy (Kerzendorfer *etal*., 2015) to the context of multiple matched samples. This capability makes thesaurus filtering now available for problems such as detection of somatic mutations in cancer genomes and *de novo* mutations in family trios. We implemented software suitable for these applications, proposed a package to handle thesaurus annotations within the R environment, and performed a number of tests on synthetic and actual data. All the tests indicated that thesaurus annotation provides a real benefit for describing changes in DNA in low mappability regions of the human genome. The improvements are observed relative to existing methods that report mutations at individual genomic loci through single entries in VCF files rather than clusters of loci.

The contribution of this work is twofold. First, the software proposes a particular implementation of mutation calling in matched samples, i.e. an extension of existing analysis pipelines incorporating thesaurus annotation. In this direction, we showed that the software is compatible with existing workflows including different aligners and variant callers. Second, our work provides tools and data structures upon which specialized applications can draw on. The formats are intentionally generic to enable extensions and adaptations of thesaurus annotations to particular needs. In particular, selection of mutation candidates can be adjusted in many ways beyond the filters described here.

A practical conclusion of the benchmarking studies is that thesaurus annotation opens the analysis window on between five and ten percent of the human genome. But the thesaurus technique also has some limitations. The implementation used in this work is not adapted to the study of indels or variants immediately near structural rearrangements. Also, because a signal from one locus is often diluted over multiple genomic sites because of alignment ambiguity, the coverage required to detect and call a variant or mutation in low mappability regions is higher than in high mappability regions. With decreasing sequencing costs and gradually increasing standards of sample coverage and read length, however, this factor should not be a serious obstacle to the study of mutations in these regions.

## Supplementary Material

Supplementary Data
