# Supplementary material for: Comparison of genetic variants in matched samples using thesaurus annotation
Source: Bioinformatics. 2015 Nov 5;32(5):657–63. doi: 10.1093/bioinformatics/btv654 (PMC4795618; doi:10.1093/bioinformatics/btv654)
Supplement: Supplementary Data [file btv654_supplementary_data.zip › thesMS_supp.pdf]

Supplementary Text: Comparison of genetic variants in matched samples using thesaurus annotation

T. Konopka[1,*], and S.M.B. Nijman[1,*]

[1] *Ludwig Institute for Cancer Research, University of Oxford, Oxford, UK*

[*] *Authors in alphabetical order*

This Supplementary Text contains methods and results to accompany the manuscript of the same name.

Contents

1 Software **2**
- 1.1 GeneticThesaurus - java . . . 2
- 1.2 GeneticThesaurus - R . . . 2

2 Methods **2**
- 2.1 Synthetic Data . . . 2
- 2.2 Running thesaurus annotation . . . 2
- 2.3 Measurement of Performance . . . 2

3 Results - Paired Samples **2**
- 3.1 Random Links . . . 2
- 3.2 Mutect . . . 4
- 3.3 Varscan2 . . . 5
- 3.4 Platypus . . . 6

4 Results - Family Trio **7**
- 4.1 Robustness of thesaurus annotation . . . 7

5 Platinum Family Trio **9**
- 5.1 Comparison with long-insert data . . . 9

References **11**

1 Software

The software developed for this work has two main parts. The first is variant annotation, performed with a java program GeneticThesaurus. The second is mutation calling and sample meta-analysis, performed in R [1] with package RGeneticThesaurus and other custom scripts.

1.1 GeneticThesaurus - java

The java program GeneticThesaurus (sourcefore: `http://sourceforge.net/projects/geneticthesaurus/`; github: `https://github.com/tkonopka/GeneticThesaurus`) annotates called variants with thesaurus links. This program looks through variants defined in a vcf file and determines a network of thesaurus links based on an alignment and a thesaurus file [2]. The version used in this work was 0.2.0. For this version, we implemented important features that enable the software to scan multiple alignment files (e.g. matched normal and tumor samples) extracting allelic frequencies for each variant from each of the alignments.

1.2 GeneticThesaurus - R

After annotating variants, we performed mutation calling and mutation comparison within the R environment [1]. A package including all the relevant functions is available on github `https://github.com/tkonopka/RGeneticThesaurus`.

2 Methods

To evaluate various methods for detecting mutations - with and without thesaurus annotations - we used synthetically generated samples and performance metrics suited to the thesaurus setting.

2.1 Synthetic Data

For benchmarks with two matched samples (normal/tumor pairs) we created synthetic data as follows. We generated one genome based on hg38 with randomly placed substitutions at a rate of $1 \cdot 10^{-3}$ bp^{-1} (one substitution per kilobase). We create a second genome by further mutating the first genome at a rate of $0.5 \cdot 10^{-3}$ bp^{-1}. These constitute the normal and tumor genomes.

We then created 20x coverage samples from the normal genome and from the tumor genome. We also created samples representing mixtures of these two at various allelic proportions. For each sample, we generated paired-end 100bp reads with an insert size of 400bp; the tool for read generation is available through the GeneticThesaurus java program.

2.2 Running thesaurus annotation

We ran the GeneticThesaurus annotation tool with the following parameters: `--minmapqual` set to the MQ values described in the analyses, `--insertsize 400`, `--many 50`, and `--toomany 100`. The last two settings limit the number of alternative sites listed in association with annotated variants.

2.3 Measurement of Performance

To measure performance of various mutation calling methods, we used definitions for true positive rate and false discovery rate as explained in the main text.

3 Results - Paired Samples

The purpose of this section is to compare mutation detection approaches on the first synthetic dataset described in the methods. The dataset consists of paired normal/tumor samples, wherein the tumor sample contains simulated somatic single-nucleotide substitutions.

3.1 Random Links

In a first comparison, we called mutations in the synthetic data with Bamformatics (v0.2.3) (`http://sourceforge.net/projects/bamformatics/`) using two mapping quality thresholds, MQ1 and MQ16. We then annotated the variants with the genetic thesaurus [2] and called mutations based on changes

in allelic frequency. As a control, we also called mutations based on raw variant calls and annotated them with a random set of 'thesaurus' links. The results are summarized in Figure S1.

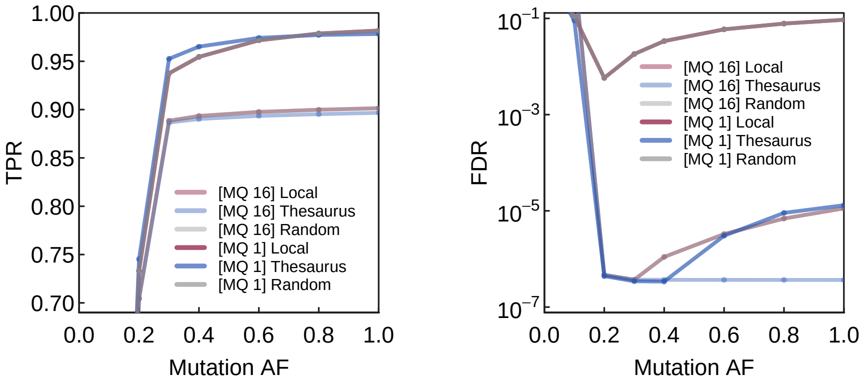

Figure S1: **Mutation detection performance with thesaurus annotation.** (Left) Recall of known mutations from synthetic samples whit different mutation allelic frequency (AF) (Right) False discovery rate for mutations detected in the same synthetic samples. High apparent FDR at $AF < 0.2$ is a consequence of our definition of FDR which greatly penalizes this metric when the total number of calls made is very small. (Both panels) Light color is used for calculations based on stringent mappability threshold, where thesaurus annotation does not provide much benefit. Dark color is used for calculations including candidates from low mappability regions; thesaurus annotation reduces the false positive rate by orders of magnitude.

In samples where mutations were present at very low allelic frequency, very few mutations were detected regardless of mappability thresholds. At higher allelic frequencies, all methods recovered a substantial number of actual positives.

In terms of recall, low mapping quality threshold (MQ1) gave substantially better performance than the high threshold (MQ16). Thesaurus annotation changed the recall curves slightly, notably for mutations at intermediate alellic frequencies. In contrast, random links did not substantially increase performance. (In the figure, gray lines representing performance with random links almost exactly overlap the red lines corresponding to the local analysis.)

In terms of false discovery rate, we observed that the high mapping quality threshold kept false positives very low, as expected. Thesaurus annotation reduced this further. At low mapping quality thresholds, in the traditional analysis, the false discovery rate was over three orders of magnitude higher. With the thesaurus annotation, the false discovery rate dropped to levels comparable to the high-mapping quality settings. Notably, comparable drops in FDR were not observed as a result of random thesaurus links.

3.2 Mutect

We also compared our mutation calls based on allele-frequency to available tools. The first alternative tool that we tried was Mutect (v1.1.4-28) [3] - a tool designed for analysis of cancer genomes (Figure S2).

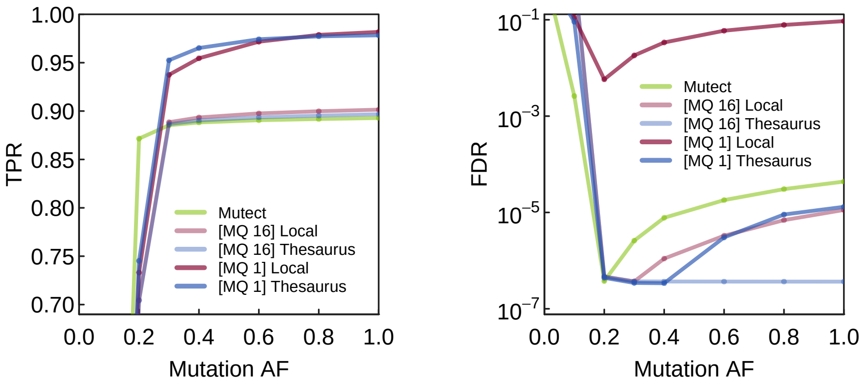

Figure S2: **Mutation detection performance with Thesaurus.** Analogous to figure S1. Here mutation calls with and without thesaurus annotation are compared to MuTect, a popular mutation detection program. MuTect filters variants by mappability, thereby ignoring mutations in low mappability regions.

On our synthetic dataset, MuTect performed similarly to our high mapping quality setting (Figure S2). The Mutect recall curve was slightly better than ours at low allelic frequencies, and the false discovery rate was only marginally higher. Such a trade-off in performance can be attributed to slightly different default thresholds. Overall, however, the default settings in Mutect closely matched our own thresholds in the our custom mutation calling procedure.

Importantly, after thesaurus annotation, calls based on allele-frequency changes gave higher recall and lower false discovery rate than the base Mutect calls.

3.3 Varscan2

In a second comparison, we tried Varscan2 (v2.3.7) [4] - another somatic mutation caller designed for analysis of cancer genomes. This program accepts filtering based on mappability through samtools, so we produced mutation calls for both MQ1 and MQ16 settings for all our samples (Figure S3).

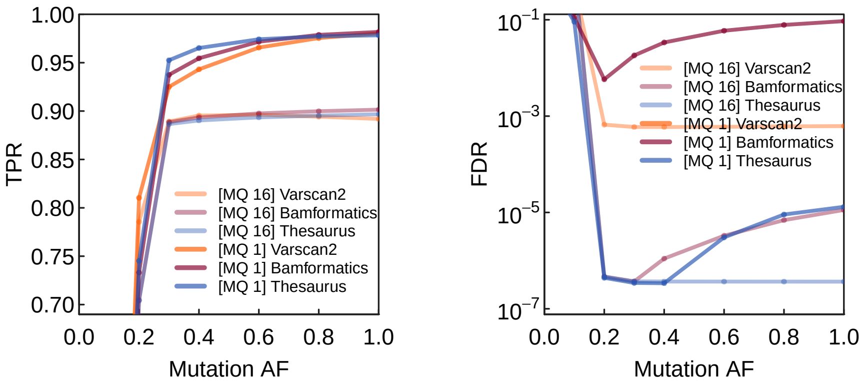

Figure S3: **Mutation detection performance with thesaurus annotation.** Analogous to figure S1. Here mutation calls with and without thesaurus annotation are compared to Varscan2, a popular mutation detection program. Varscan2 shows high false positive rate in all the samples, possibly due to misclassification of germline variants as somatic mutations.

The recall curves from Varscan2 followed closely the two curves produced with our approach. The false discovery rate curves, however, were substantially different. Unsurprisingly, the Varscan false positive rate curve was higher at MQ1 than at MQ16. But it is difficult to make further comparisons as Varscan2 produced quite a large number of false positives, even in samples with low or zero tumor content. These were likely germline mutations in our synthetic samples that were misclassified as mutations. In principle it should be possible to remove such germline contamination from the Varscan2 results, so this should not be considered as an overly negative result. However, we did not pursue this germline filtering strategy in detail here.

We note that Varscan2 can be viewed as a representative of a larger class of approaches that use input from samtools mpileup. Thus, the improvements in mutation calling brought forth by thesaurus annotation is extendible to most of these alternative approaches as well. That is because the improvements in limiting false discovery are due to linking multiple sites together, regardless of the details of processing local information to filter-in or filter-out individual sites.

3.4 Platypus

Finally, we tried Platypus [5]. Platypus is a variant caller and does not in itself produce mutation calls from paired samples, so we did not evaluate it as we did Mutect or Varscan. Instead, we treated calls from Platypus in the same way as we did calls from Bamformatics in section 3.1. In other words, we annotated Platypus variants with the thesaurus software, and called mutations at sites where thesaurus-evaluated allelic frequencies differed between samples. We then compared high/low mappability thresholds as in section 3.1 (Figure S4).

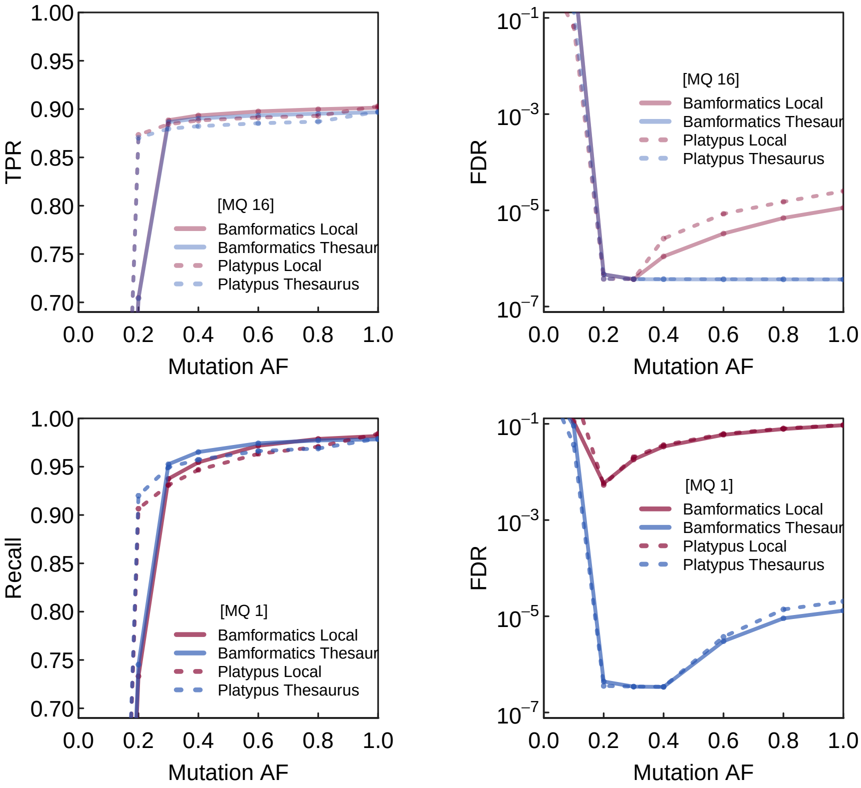

Figure S4: **Mutation detection performance with Thesaurus.** Analogous to figure S1. Here the underlying variants annotated by the genetic thesaurus are produced by Platypus, a fast and memory efficient variant caller. These results show that the thesaurus software can annotate variants and mutations from this tool just as well as variants from Bamformatics.

At MQ16 (top row in figure), the mutations detected through Platypus showed similar recall and false positive patterns as mutations detected through Bamformatics. A small difference was that the Platypus workflow provided more hits at low allelic frequencies. This reflects differences in minimum evidence thresholds in the underlying variant caller, which can be tuned in both Platypus and Bamformatics.

Also at MQ1 (bottom row in figure) mutations detected through Platypus followed similar patterns as before. The default Platypus output again yielded slightly more hits at low allelic frequencies; differences at high allelic frequencies were minimal.

These results show that the thesaurus software accepts inputs from multiple callers (in this example, Bamformatics and Platypus) and improves mutation calling performance in each case.

4 Results - Family Trio

The purpose of this section is to provide additional results for de novo mutation detection on the synthetic family trio dataset.

4.1 Robustness of thesaurus annotation

The main text describes mutation calling performance obtained with our 'standard' settings for thesaurus annotation, in particular using options `--many 50` and `--toomany 100`. These figures imply that we obtained at most 100 thesaurus links for each annotated variant. To determine whether this arbitrary threshold affects overall performance, we re-ran the annotation with more strict settings `--many 5` and `--toomany 10`, limiting the number of thesaurus links to just ten.

The main effect of this change is to change the status of several variants from filter code `thesaurus` (meaning a variant is annotated with links to alternative sites) to `thesaurusmany` (indicating that a variant can be linked to more alternative sites than is specified by the user). Our mutation calling procedure applies a hard filter on sites marked as `thesaurusmany` and `thesaurushard`, so this change of settings effectively removed these sites from the mutation hit list. As several of these are true positives or thesaurus true positives, asking for smaller thesaurus clusters effectively increased the number of false negatives. The overall performance patterns, however, were relatively similar to what we observed at our standard settings (Figure S5 and Tables S1, S2). This shows that thesaurus annotation is quite robust to changes in settings.

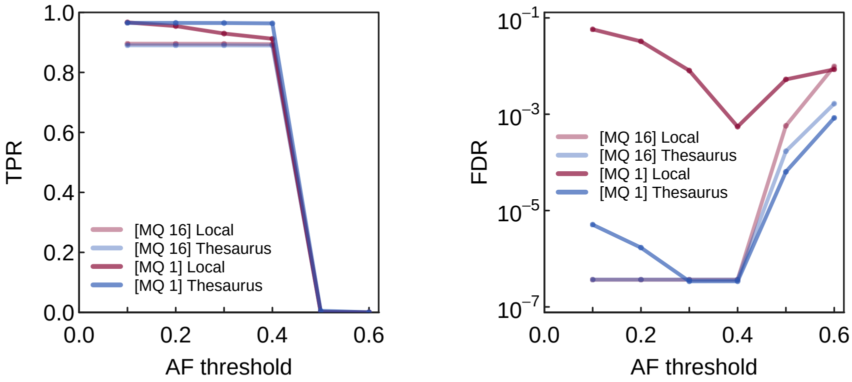

Figure S5: **Mutation detection performance in synthetic family trio with strict thesaurus settings.** Analogous to figure 3 in main manuscript, but here the underlying thesaurus link set was produced with strict settings `--many 5 --toomany 10`, allowing at most 10 links per site.

Method	MQ	BAF	TP	TTP	FP	FN
naive	16	0.1	1365192	0	0	158966
naive	16	0.2	1365072	0	0	159086
naive	16	0.3	1364619	0	0	159539
naive	16	0.4	1362900	0	0	161258
naive	16	0.5	868	0	0	1523290
naive	16	0.6	50	0	0	1524108
theslink	1	0.1	1456721	81189	8	43981
theslink	1	0.2	1456583	81185	2	44094
theslink	1	0.3	1455947	81122	0	44678
theslink	1	0.4	1454039	80551	0	46791
theslink	1	0.5	10937	3591	0	1510770
theslink	1	0.6	1053	249	0	1522909

Table S1: **Summary of mutation calling performance using settings `--many 50 --toomany 100`.** Column marked method and MQ indicate how the mutations were called, using the raw/naive variant calls and allelic frequencies, or using thesaurus filter codes, allelic frequencies, and links. BAF: threshold for allelic frequency. MQ: mapping quality threshold. TP, FP, FN: absolute numbers of true positives, false positives, and false negatives.

Method	MQ	BAF	TP	TTP	FP	FN
naive	16	0.1	1365192	0	0	158966
naive	16	0.2	1365072	0	0	159086
naive	16	0.3	1364619	0	0	159539
naive	16	0.4	1362900	0	0	161258
naive	16	0.5	868	0	0	1523290
naive	16	0.6	50	0	0	1524108
theslink	1	0.1	1449339	77765	7	53273
theslink	1	0.2	1449205	77763	2	53381
theslink	1	0.3	1448687	77706	0	53847
theslink	1	0.4	1447065	77232	0	55610
theslink	1	0.5	6403	2217	0	1516326
theslink	1	0.6	543	111	0	1523564

Table S2: **Summary of mutation calling performance using settings `--many 5 --toomany 10`.** Analogous to previous table.

5 Platinum Family Trio

The publicly available data for the Illumina Platinum Genomes datasets were obtained through the European Nucleotide Archive studies ERP001960 (short-insert) and ERP002490 (long-insert).

5.1 Comparison with long-insert data

The Platinum series contains 17 samples sequenced with short-insert technology, and a subset sequenced with long-insert technology. Paired reads in the long-insert samples are 100 bp each, but are separated by inserts of a few kilobases. This is meant to reduce mapping ambiguity, and thus reduce the impact of mapping quality on variant analysis. The coverage of these samples is around two-thirds of the short-insert analogs, and because coverage is an important determinant for variant calling, we do not present a direct comparison of called variants here. Instead, we obtained mutation calls only from the higher-coverage short-insert data and then computed the allelic frequencies on these target sites in the long-insert data (Figure S6).

Overall, the allelic frequencies in the two datasets are consistent, i.e. mutation candidates identified in the short-insert data have non-zero allelic frequencies in the long-insert data. As in the main text, the pattern for the mutation sites identified through the thesaurus approach is more noisy. Some sites identified through the thesaurus approach appear missing in the long-insert data, but the same is true for the variants in the high mapping quality regions.

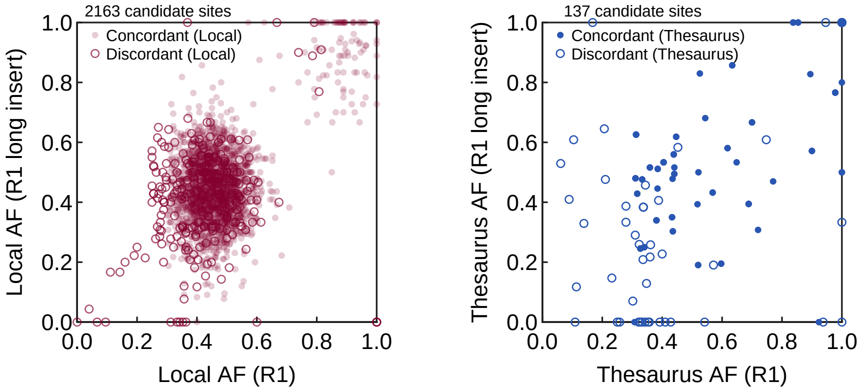

Figure S6: **Allelic frequencies on mutation candidate sites in short-insert and long-insert datasets.** (Left) High-mapping quality mutation candidates identified in the short insert-data are plotted as red points. The axes indicate allelic frequencies in the the short-insert and the long-insert data. (Right) Low mapping quality mutation candidates identified in the short-insert data are plotted as blue points. The axis show thesaurus adjusted allelic frequencies in the short- and long-insert data. For both axes, the thesaurus links used to compute adjusted allelic frequencies are those computed from the short-insert dataset. (Both panels) Concordance and discordance in both panels are between the two replicates of short-insert samples. (Both panels) Dots in the lower-left corner with very low allelic frequencies in both datasets correspond to candidates identified in the second replicates of the short-insert data.

An example of concordant variants appears in Figure S7. In this example, two mutation candidates appear in both replicates of the short-insert data and in the long-insert sample as well. The candidates are not present in either the short-insert nor the long-insert data from the father's DNA. As the candidates are on chromosome Y, coverage in the mother's samples are zero (not shown). Both candidates appear to be heterozygous, which is unexpected given that chromosome Y is haploid. The naive read count is thus deceiving.

Using thesaurus annotation, the two variants are connected via a thesaurus link. The two sites are therefore understood to be represent a single alteration in the child's genome with allelic frequency closer to unity that would appear from the diagrams. In other words, the thesaurus-assisted interpretation of the data is that the child's genome contains a single mutation that is manifest at two sites in the reference genome.

Of note is that the mapping ambiguity in the short-insert data is also present in the long-insert data. In the long-insert datasets, reads aligned to both sites have low mapping quality. In some cases, this is because reads are not paired (one mate is unmapped), but there are also several example where reads have inserts between 2kb and 5kb. For these mutations, and by manual inspection most other candidates in the Platinum trio, long-insert sequencing is not capable of resolving mutations in sequence-similar regions.

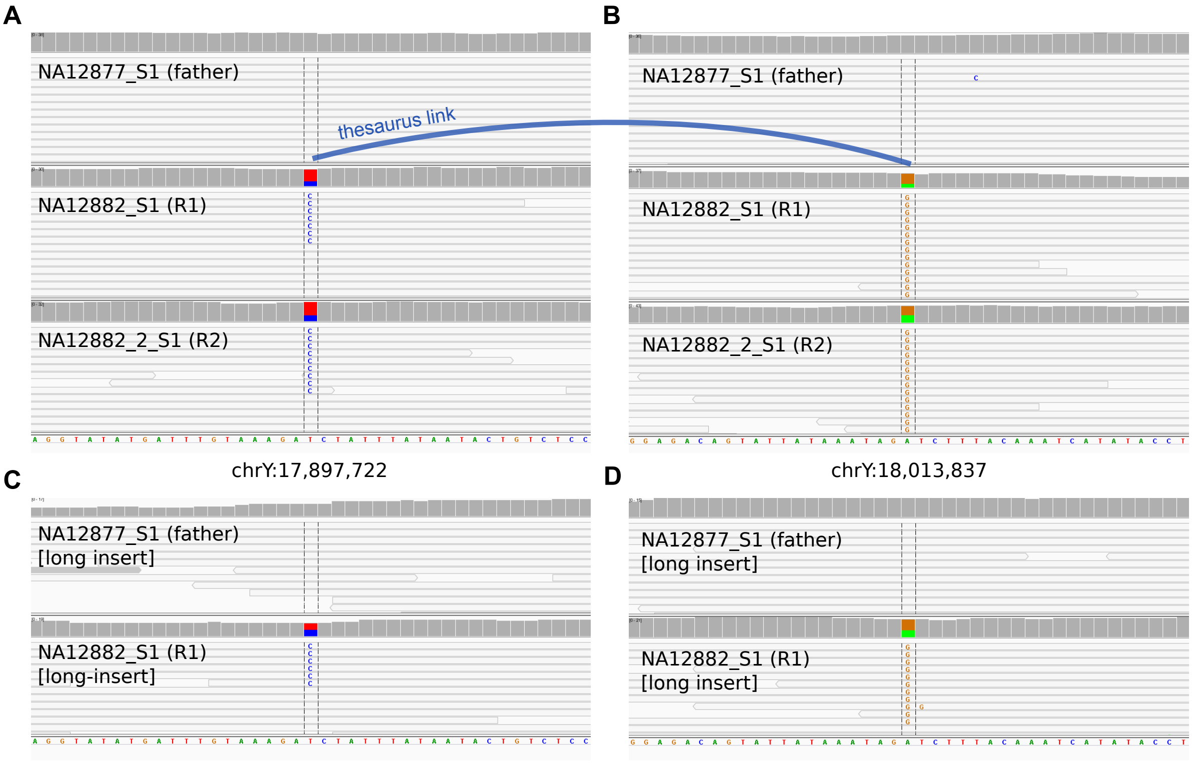

Figure S7: **An example of a mutation candidate in short-insert and long-insert datasets.** All panels are screen-shots from IGV with alignments of the Platinum series; white boxes represent reads with low mapping quality. (A) Three short-insert samples showing a called mutation on chromosome Y. The top alignment shows the father's genome with no evidence for the variant. The other two alignments display evidence for the variant in two replicates of the child's genome. (B) Another called mutation on chromosome Y, linked to the first via a thesaurus link. (C) Analogous to panel A, but here showing reads from the long-insert samples. (D) Analogous to panel B, but here showing reads from the long-insert samples.

References

[1] R Core Team, *R: A Language and Environment for Statistical Computing.* R Foundation for Statistical Computing, Vienna, Austria, 2014.

[2] C. Kerzendorfer, T. Konopka, and S. M. Nijman, "A thesaurus of genetic variation for interrogation of repetitive genomic regions," *Nucleic Acids Research*, p. gkv178, 2015.

[3] K. Cibulskis, M. S. Lawrence, S. L. Carter, A. Sivachenko, D. Jaffe, C. Sougnez, S. Gabriel, M. Meyerson, E. S. Lander, and G. Getz, "Sensitive detection of somatic point mutations in impure and heterogeneous cancer samples," *Nature biotechnology*, vol. 31, no. 3, pp. 213–219, 2013.

[4] D. C. Koboldt, Q. Zhang, D. E. Larson, D. Shen, M. D. McLellan, L. Lin, C. A. Miller, E. R. Mardis, L. Ding, and R. K. Wilson, "VarScan 2: somatic mutation and copy number alteration discovery in cancer by exome sequencing," *Genome Research*, vol. 22, no. 3, pp. 568–576, 2012.

[5] A. Rimmer, H. Phan, I. Mathieson, Z. Iqbal, S. R. Twigg, A. O. Wilkie, G. McVean, G. Lunter, W. Consortium, *et al.*, "Integrating mapping-, assembly-and haplotype-based approaches for calling variants in clinical sequencing applications," *Nature genetics*, vol. 46, no. 8, pp. 912–918, 2014.
